# Non-adherence in non-inferiority trials: pitfalls and recommendations

**DOI:** 10.1136/bmj.m2692

**Published:** 2020-07-10

In this paper by Mo and colleagues (*BMJ* 2020;370:m2215, doi:10.1136/bmj.m2215, published 1 July 2020), the funding statement was incomplete and should have included the following sentence in full: “BSC is supported by UK Medical Research Council/Department for International Development (grant MR/K006924/1) and the UK Department of Health and Social Care using UK Aid funding managed by the National Institute for Health Research (grant PR-OD-1017-20006).” Furthermore, in the summary box, the third point should read: “Potential confounders should be prespecified in order to collect relevant and complete data from both adherent and non-adherent participants during the trial.”

